# A shared metabolic-immune axis links local and systemic inflammation in chronic rhinosinusitis with comorbid asthma

**DOI:** 10.3389/falgy.2025.1700350

**Published:** 2026-01-29

**Authors:** Xin Peng, Zhili Li, Yibo Liang, Guimin Zhang

**Affiliations:** 1 Department of Otorhinolaryngology Head and Neck Surgery, Tianjin First Central Hospital, Tianjin, China; 2 Department of Otorhinolaryngology Head and Neck Surgery, Tianjin First Central Hospital, Institute of Otolaryngology of Tianjin, Tianjin, China; 3 Key Laboratory of Auditory Speech and Balance Medicine, Tianjin, China; 4 Key Medical Cultivation Discipline of Tianjin (Otolaryngology), Tianjin, China; 5 Quality Control Centre of Otolaryngology, Tianjin, China

**Keywords:** asthma, chronic rhinosinusitis with nasal polyps, lipid metabolism, metabolic-immune axis, metabolomics

## Abstract

**Background:**

Chronic rhinosinusitis with nasal polyps (CRSwNP) comorbid with asthma (CRSwA) represents a severe “unified airway” phenotype, yet the metabolic mechanisms linking upper and lower airway inflammation remain unclear. This study aimed to identify shared metabolic signatures connecting local pathology with systemic circulation by comparing the metabolic profiles of nasal polyp tissue and serum.

**Methods:**

We performed an integrated analysis using non-targeted metabolomics and transcriptomics on paired nasal polyp tissue and serum samples from 22 CRSwA patients and 40 non-asthmatic CRSwNP patients to identify differential metabolites and explore their association with the immune microenvironment.

**Results:**

CRSwA patients exhibited distinct metabolic signatures dominated by lipids and their derivatives in both tissue and serum. An analysis of the metabolome shared between compartments revealed a weak but significant positive correlation in metabolic fold changes, suggesting a subtle systemic link to the local inflammation. This shared metabolic profile was strongly associated with a local Th2-polarized immune microenvironment. This shared profile was strongly associated with a local Th2-polarized immune microenvironment, where key metabolites (e.g., Resolvin D2, Lipoxin A4) correlated significantly with the abundance of M2 macrophages and eosinophils. Furthermore, a logistic regression model based on serum metabolites effectively distinguished CRSwA from non-asthmatic CRSwNP (AUC = 0.8322).

**Conclusion:**

Our study reveals a highly conserved “metabolic-immune axis” that connects local tissue inflammation with systemic circulation, positioning metabolic dysregulation as a central hub in the unified airway disease model for CRSwA. These findings offer new perspectives for developing serum-based diagnostic markers and metabolically-targeted therapies for this challenging clinical condition.

## Introduction

Chronic rhinosinusitis (CRS) is a complex chronic inflammatory disease, among which chronic rhinosinusitis with nasal polyps (CRSwNP) represents a particularly recalcitrant phenotype. Its well-established comorbidity with asthma is of significant clinical interest, as both conditions are archetypal Type 2 inflammatory diseases characterized by the prominent upregulation of cytokines such as IL-4, IL-5, and IL-13 (1, 2). This shared inflammatory signature is closely associated with increased disease severity, recurrence rates, and therapeutic resistance (3, 4). Mounting evidence indicates a significant correlation between nasal and lower airway inflammation in patients with CRSwNP, lending strong support to the “unified airway” hypothesis, which posits that the upper and lower airways are immunologically and inflammatorily interconnected (5, 6). This association suggests that the comorbidity of CRSwNP and asthma may involve deeper, shared pathophysiological mechanisms, particularly the dysregulation of local and systemic metabolism.

In recent years, metabolomics has emerged as a powerful tool for elucidating disease mechanisms (7). Through the systematic analysis of small-molecule metabolites, this approach can uncover disease-associated perturbations in metabolic pathways and identify potential biomarkers (8). Within the context of CRSwNP, it is hypothesized that localized sinonasal inflammation can trigger systemic metabolic alterations; conversely, systemic metabolic dysregulation may exacerbate the local inflammatory response. For instance, inflammatory mediators released from the nasal mucosa may not only modulate local metabolism but could also enter systemic circulation, thereby influencing the lower respiratory tract and establishing a metabolic crosstalk between the upper and lower airways (9). This interplay between local and systemic metabolism offers a novel framework for understanding the pathogenesis of comorbid CRSwNP and asthma.

To address this knowledge gap, the present study was designed to compare the metabolic profiles of nasal polyp tissue and serum from CRSwNP patients. By identifying metabolites that show congruent changes in both compartments, we aimed to uncover the metabolic signatures that could serve as a bridge between localized upper airway inflammation and systemic responses, thereby providing new insights into the pathophysiology of the unified airway.

## Materials and methods

### Patient enrollment and sample collection

This single-center, cross-sectional study was conducted at the Tianjin First Central Hospital between June 2020 and July 2022, following approval from the institution's Ethics Research Committee. All participants provided written informed consent prior to enrollment. The sample size was determined *a priori* to ensure sufficient statistical power. Based on a desired power of 80% to detect a large, biologically meaningful effect size (Cohen's d = 0.8) at a significance level of 0.05, a minimum of 19 patients with comorbid asthma (CRSwA) and 33 non-asthmatic CRSwNP patients was required. We ultimately enrolled a cohort of 22 CRSwA and 40 non-asthmatic CRSwNP patients (total *N* = 62), which exceeds the calculated minimum and confirms that the study was adequately powered to robustly identify metabolic signatures. All CRSwNP diagnoses were established according to the European Position Paper on Rhinosinusitis and Nasal Polyps (2020) criteria, while asthma diagnoses were based on the Global Initiative for Asthma (GINA) guidelines, with all asthmatic patients demonstrating stable symptom control for at least one year (10, 11). Baseline respiratory assessments were confirmed via lung function tests for all participants before surgery.

Demographic and clinical information, such as age, sex, total serum immunoglobulin E (IgE) levels, Sino-Nasal Outcome Test-22 (SNOT-22), visual analog scale (VAS) scores, and Lund-Mackay CT (LM) scores, were carefully recorded, with all diagnoses confirmed independently by two skilled rhinologists to ensure precision. To preserve the integrity of the study cohort, patients with immune disorders, genetic diseases, cystic fibrosis, unilateral polyposis, acute infections, anatomic sinusitis, or recent (<12 weeks) use of antibiotics, immunosuppressants, or corticosteroids (systemic or topical) were excluded. Surgical interventions followed established protocols, and tissue specimens were collected for further sequencing and analysis.

Paired nasal polyp tissue and serum samples were processed under a standardized protocol to ensure biomolecular integrity. Freshly resected nasal polyp tissues, handled under RNase-free conditions, were immediately rinsed with PBS, trimmed into sub-5 mm fragments, and flash-frozen in liquid nitrogen vapor. Concurrently, preoperative venous blood was processed within 60 min, undergoing a two-step centrifugation (1,800 × g for 10 min; 13,000 × g for 2 min) to yield particulate-free serum. All tissue and serum aliquots were stored at −80 °C and transported on dry ice. This rigorous protocol was designed to minimize pre-analytical variability, guaranteeing high-quality, stable samples for subsequent transcriptomic and metabolomic analyses.

### Integrated transcriptomic and immune profiling of nasal polyp tissues

To explore gene expression patterns, total RNA with high integrity (RIN > 7.0, concentration >50 ng/μL) was isolated and subjected to poly(A) mRNA enrichment. The RNA fragments were heat-treated (94 °C, 5–7 min) to construct strand-specific cDNA libraries. During reverse transcription, dUTP was incorporated to selectively inhibit second-strand synthesis, followed by paired-end sequencing on the Illumina NovaSeq 6,000 platform (LC-Bio, Hangzhou). Raw sequencing reads were filtered for quality (Cutadapt), mapped to the GRCh38 genome (HISAT2), and quantified for transcripts using StringTie. Differential expression of protein-coding genes was analyzed based on FPKM-normalized counts, revealing significant transcriptional differences across groups. In parallel, the immune microenvironment was characterized through advanced bioinformatics tools. Single-sample gene set enrichment analysis (ssGSEA) was employed to assess pathway activation scores for T-helper subsets (TH1, TH2, and TH17/TH22), using curated gene markers from published studies (12, 13). Additionally, CIBERSORT was applied to quantify 22 distinct immune cell types, providing insights into immune cell composition and spatial distribution within the tissue. To mitigate sequencing batch effects, libraries were randomized across lanes, and technical covariates (library preparation batch, lane) were recorded. Normalization employed TMM, and batch covariates were included in downstream models. PCA and surrogate variable analysis (SVA) were used to identify and adjust residual technical variation.

### Metabolite extraction and LC–MS/MS analysis

Frozen samples were thawed on ice and extracted by adding prechilled 50% methanol at a 1:6 sample-to-solvent ratio (w/v), followed by vortex mixing and overnight incubation at −20 °C. After centrifugation at 4,000 × g for 20 min, the supernatant was subjected to LC–MS/MS analysis. Chromatographic separation was performed on a Thermo Fisher Scientific UltiMate 3,000 UPLC system using a Waters ACQUITY UPLC T3 column (100 mm × 2.1 mm, 1.8 µm) maintained at 40 °C. The mobile phases were solvent A (water with 5 mM ammonium acetate and 5 mM acetic acid) and solvent B (acetonitrile), at 0.3 mL/min. The gradient was: 0–0.8 min, 2% B; 0.8–2.8 min, 2% → 70% B; 2.8–5.6 min, 70% → 90% B; 5.6–6.4 min, 90% → 100% B; 6.4–8.0 min, 100% B; 8.0–8.1 min, 100% → 2% B; 8.1–10.0 min, 2% B. Mass spectrometry used a Thermo Fisher Scientific Q Exactive Orbitrap with an electrospray ionization source in both positive and negative modes. The spray voltages were 3.5 kV (positive) and −2.8 kV (negative), capillary temperature 320 °C, and sheath/aux gas 35/10 (instrument units). Full scans were acquired over m/z 70–1,050 at 70,000 resolution (@ m/z 200), AGC target 3 × 10^6^, and maximum injection time 100 ms. Data-dependent acquisition (DDA) used a top-3 method; MS/MS spectra were acquired at 17,500 resolution, AGC target 1 × 10^5^, maximum injection time 80 ms, with normalized collision energies of 20, 40, and 60 eV. Sample injection order was randomized; pooled QC samples were injected every 8–10 study samples; the instrument was calibrated prior to each batch.

### Raw data processing and feature identification

LC–MS raw files were converted to mzXML and processed in R using XCMS for peak picking, retention time correction, alignment, and feature grouping, followed by CAMERA for annotation of isotopes, adducts, and potential in-source fragments. Preprocessing with metaX included QC-based signal drift correction using LOESS, probabilistic quotient normalization (PQN) to account for sample-wise dilution, and k-nearest neighbors imputation for sporadic missing values. Features with >30% missing values in either clinical group were removed prior to statistics. Analytical quality control required tight QC clustering in PCA and a median relative standard deviation (RSD) of QC features <20%. Batch covariates were recorded and considered in downstream modeling.

### Metabolite annotation and identification

Each ion feature was defined by paired retention time (RT) and m/z. Annotation used exact mass matching against HMDB and KEGG; features with mass error <10 ppm were retained as candidates and further verified by isotopic distribution and adduct relationships provided by CAMERA. Where available, MS/MS spectra were matched against HMDB, KEGG, and an in-house library using a spectral similarity score >70, and inspected for diagnostic fragment ions when applicable. Identification confidence followed the Metabolomics Standards Initiative (MSI): Level 2 (putatively annotated compounds) required accurate mass (<10 ppm), isotope/adduct consistency, and supportive MS/MS similarity; Level 3 (putatively characterized compound classes) was assigned when class-level features were present without unique structure-specific MS/MS. For lipids, acyl chain composition is reported when fragment ions support it; sn-position and double-bond localization were not assigned.

### Integrated analysis of metabolite and immune interactions

The study focused on identifying metabolites that exhibited consistent directional changes—either increased or decreased—in both serum and tissue samples from CRSwNP patients compared to healthy controls. These conserved metabolites were further analyzed to explore their relationships with immune cell populations and T-helper cell pathway activities. Tissue-specific and serum-specific metabolites were independently assessed using Spearman's correlation to uncover compartment-specific metabolic-immune interactions. This stratified approach provided valuable insights into the distinct roles of metabolites in modulating immune responses within different biological compartments, enhancing understanding of the metabolic-immune crosstalk in CRSwNP pathogenesis.

### Statistical analysis

All statistical analyses were conducted in R (version 4.2.2) and GraphPad Prism (version 9.0). Normality of clinical variables was assessed by the Shapiro–Wilk test and Q–Q plots; group comparisons used Student's t-test or one-way ANOVA with Tukey's *post hoc* test for normally distributed data and Mann–Whitney U or Kruskal–Wallis tests for non-normal data, with two-sided *p*-values. Categorical variables were analyzed by Pearson's *χ*² test or Fisher's exact test as appropriate.

Multivariate analyses included PCA and PLS-DA (ropls package), with VIP > 1.0 indicating variable importance. Univariate testing used two-sided *t*-tests with FDR correction (*p* < 0.05). Final selection of differential metabolites required simultaneously meeting three criteria: *p* < 0.05, fold change >1.2 (or <1/1.2), and VIP > 1 from PLS-DA. Permutation testing was performed to evaluate overfitting risk. KEGG-based pathway enrichment used a hypergeometric test, and network visualization was constructed according to the pathways of the significant metabolites. To integrate immune information, Spearman correlations were computed between metabolite intensities and tissue-level ssGSEA pathway scores as well as CIBERSORT immune cell abundances, to interrogate metabolite–immune interactions. Cross-compartment consistency was evaluated using only metabolites that were significantly differential in both tissue and serum and changed in the same direction. We computed log2(FC) for CRSwA vs. CRSwNP and performed Pearson correlation and linear regression.

## Results

### Characteristics of study participants

Paired tissue and serum samples from 62 participants (CRSwA, *n* = 22; CRSwNP, *n* = 40) were profiled. Baseline demographics and clinical characteristics are summarized in Supplementary Table S1. There were no significant between-group differences in sex distribution, age, smoking status, prior revision surgery, allergic rhinitis prevalence, serum total IgE, VAS, SNOT-22, or Lund–Mackay CT scores (all *P* *>* *0.05*).

Regarding clinical evaluation metrics, CRSwA exhibited trends toward higher rates of allergic rhinitis (AR), elevated serum total IgE levels, higher total symptom VAS scores, and increased SNOT-22 scores compared to CRSwNP. However, these differences, along with those in LM scores, did not reach statistical significance (all *P* > 0.05). Additionally, transcriptomic sequencing was performed on nasal polyp tissue from 59 eligible participants to characterize inflammatory endotypes for further investigation. This multi-omic approach provides a comprehensive molecular characterization of chronic rhinosinusitis phenotypes, enabling deeper insights into the disease mechanism.

### Metabolic reprogramming drives the Th2-polarized immune microenvironment in CRSwA

**Figure 1:**
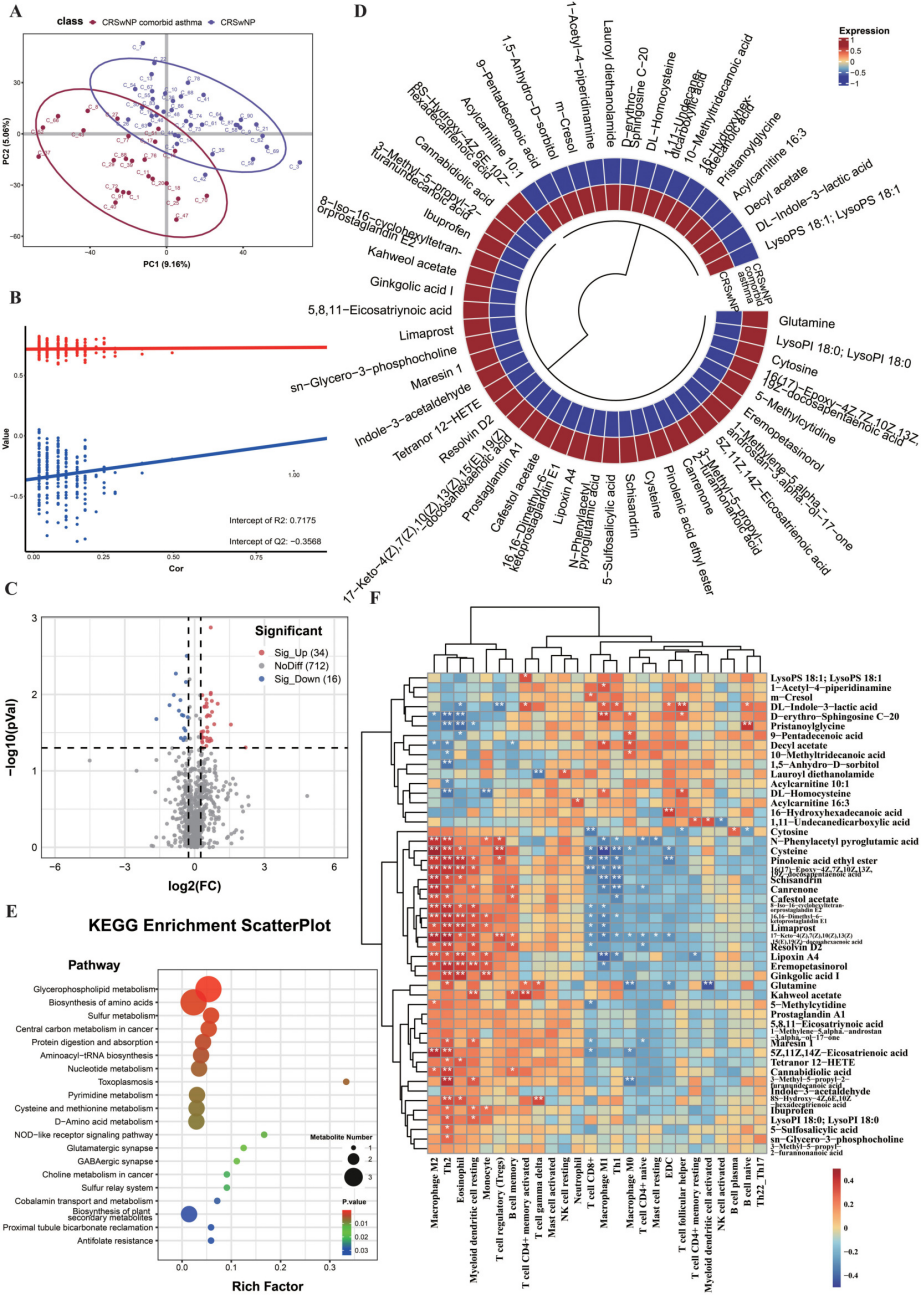
Tissue metabolomics distinguishes CRSwA from non-asthmatic CRSwNP and links lipid mediators to a Th2-polarized microenvironment **(A)** PLS-DA score plot of nasal polyp tissue metabolomes separates CRSwA (*n* = 22) from CRSwNP (*n* = 40). Pooled QC injections cluster tightly, indicating analytical stability. **(B)** Permutation testing (200 permutations) supports model robustnes. **(C)** Volcano plot of tissue features; significantly different metabolites are defined by FDR-adjusted *p* < 0.05 (two-sided *t*-test on log-transformed intensities), |fold change| ≥ 1.2, and VIP ≥ 1.0. Counts of Sig_Up and Sig_Down are indicated. **(D)** Circular heatmap of selected significant tissue metabolites (row z-scores), annotated by class. **(E)** KEGG pathway enrichment of significant tissue metabolites (hypergeometric test; bubble size denotes metabolite count per pathway; color denotes nominal *p*-value; Rich Factor shown on *x*-axis). **(F)** Spearman correlations between selected tissue metabolites and immune features (CIBERSORT cell fractions and ssGSEA pathway scores); only associations with |*ρ*| ≥ 0.3 and FDR q < 0.05 are marked. All *p*-values two-sided; multiple testing was controlled by Benjamini–Hochberg FDR at 5%.

To investigate the unique metabolic patterns in the upper airway tissue of patients with CRSwNP and comorbid asthma, we conducted a non-targeted metabolomics analysis of their nasal polyp samples. Initially, PLS-DA revealed a significant separation in the metabolic profiles between the CRSwA group and the CRSwNP without asthma group (Figure 1A), indicating distinct metabolic differences between the two cohorts. QC-based assessments demonstrated tight clustering of pooled QC samples and low RSDs after correction, supporting that group separations were not driven by batch artifacts. A subsequent permutation test (Figure 1B) confirmed the robustness and reliability of the PLS-DA model (R2 = 0.7175, Q2 = 0.3568), thereby ruling out the possibility of overfitting.

Having established significant metabolic differences between the groups, we proceeded to identify the specific metabolites responsible. A volcano plot (VIP > 1, *p* < 0.05; Figure 1C) identified 50 significantly differential metabolites, of which 34 were upregulated and 16 were downregulated in the tissue of CRSwNP patients with comorbid asthma. Chemical classification analysis (Figure 1D) indicated that lipids and their derivatives were the primary class of molecules driving these differences. Notably, Glutamine, a key molecule in energy metabolism and cell proliferation, was significantly upregulated in the CRSwA tissue, suggesting a hypermetabolic state within the local inflammatory environment. Furthermore, lipid mediators with anti-Th1 inflammatory functions, including Resolvin D2, Prostaglandin A1, and Lipoxin A4, were also significantly increased. Notably, the elevation of specialized pro-resolving mediators (SPMs) such as Resolvin D2 and Lipoxin A4, alongside their positive correlations with eosinophils and M2 macrophages, suggests a compensatory but potentially insufficient resolution response. This pattern may indicate “failed resolution” in a chronic Th2 milieu.

To contextualize these metabolic differences at the pathway level, we performed KEGG enrichment analysis on tissue differential metabolites. The enrichment scatter plot (Figure 1E) indicates significant over-representation of glycerophospholipid metabolism, biosynthesis of amino acids, sulfur metabolism and sulfur relay systems, nucleotide and pyrimidine metabolism, choline metabolism, and aminoacyl-tRNA biosynthesis, supporting coordinated reprogramming of lipid and amino acid/nucleotide metabolism in CRSwA tissue.

Building on this, we further investigated the correlation between these differential metabolites and local immune cell infiltration levels to uncover how metabolic shifts shape the immune microenvironment in CRSwA. As shown in the correlation heatmap (Figure 1F), the metabolites and immune cells exhibited significant co-variation patterns. Specifically, multiple lipid mediators that inhibit Th1 inflammation, such as Resolvin D2, Lipoxin A4, and Maresin 1, showed a significant positive correlation (*p* < 0.01) with the abundance of M2 macrophages, eosinophils, and resting myeloid dendritic cells. This indicates that these metabolites may be directly involved in the maintenance of Th2-type inflammation in CRSwA by promoting the infiltration of immunoregulatory cells and Th2-associated effector cells. Concurrently, the positive correlation between glutamine levels and both γδ T cells and CD4+ T cells (*p* < 0.05) further suggests that glutamine may indirectly promote the Th2 immune response by supporting the energy demands and functions of immune cells within the inflammatory milieu.

Collectively, these findings demonstrate that metabolic reprogramming in CRSwA not only directly regulates inflammation resolution pathways via alterations in lipid mediators but may also indirectly influence immune cell differentiation and function through the dysregulation of energy metabolism, ultimately leading to the Th2 polarization of the local immune microenvironment.

### A systemic metabolic signature in serum reflects the local immune microenvironment in CRSwA

**Figure 2:**
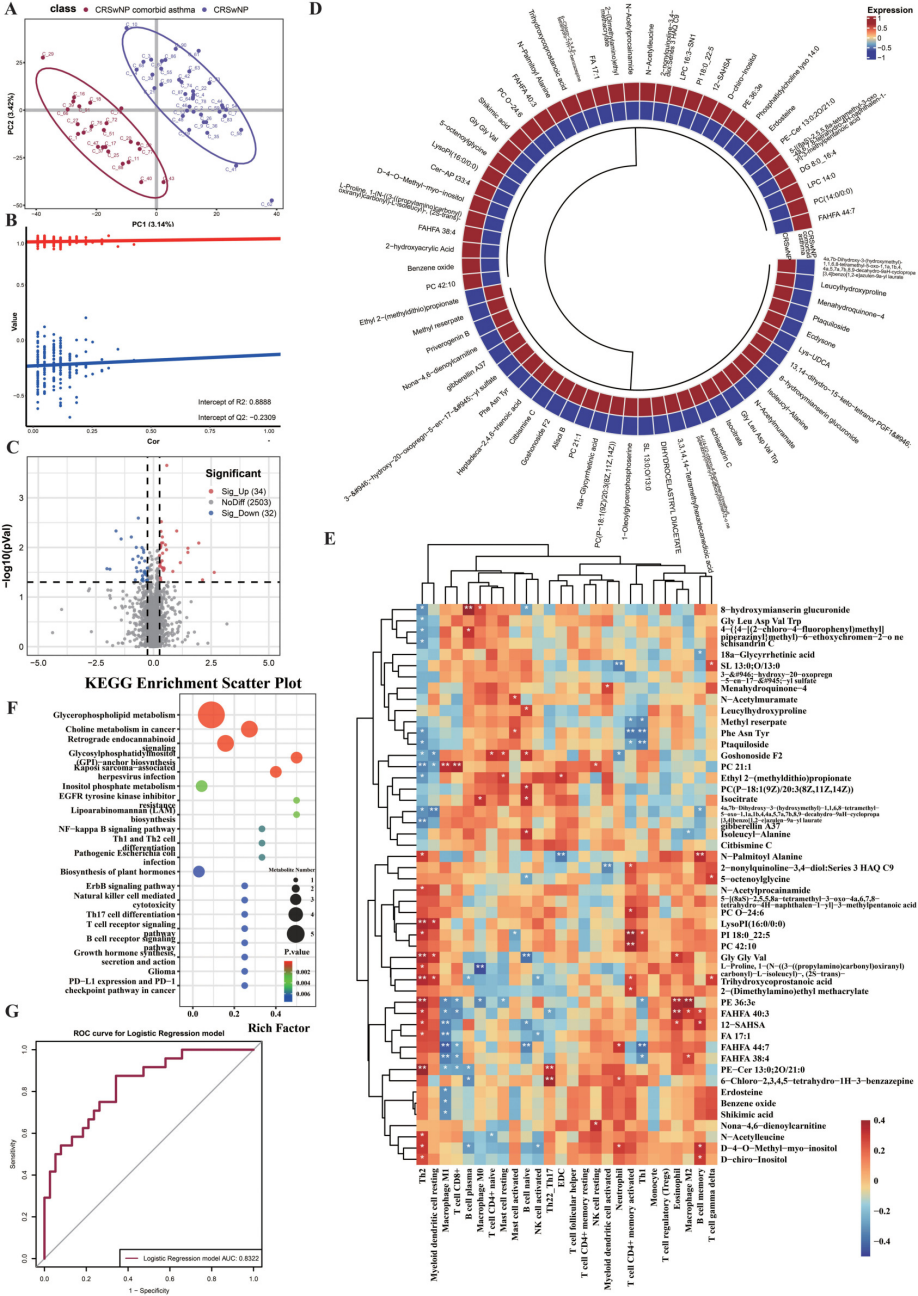
Serum metabolomics reveals a systemic lipid-centric signature and supports serum-based discrimination of CRSwA. **(A)** PLS-DA of serum metabolomes separates CRSwA (*n* = 22) from CRSwNP (*n* = 40); QC samples cluster tightly. **(B)** Permutation testing (200 permutations) indicates model robustness. **(C)** Serum volcano plot using the same significance criteria as tissue (FDR-adjusted *p* < 0.05, |fold change| ≥ 1.2, VIP ≥ 1.0); counts of Sig_Up and Sig_Down indicated. **(D)** Heatmap of key differential serum metabolites (row z-scores) with class annotations. **(E)** Correlation heatmap linking serum differential metabolites to tissue immune features; only |*ρ*| ≥ 0.3 and FDR *q* < 0.05 are highlighted. **(F)** KEGG enrichment of serum differential metabolites (bubble size = metabolite count; color = nominal *p*-value; Rich Factor on *x*-axis). G ROC curve for the logistic regression model built from FDR-significant serum metabolites; AUC = 0.8322. Performance reflects internal evaluation only; no external validation was performed. All tests two-sided; FDR controlled at 5% unless otherwise indicated.

To further investigate the systemic metabolic differences between CRSwNP patients with and without comorbid asthma, we conducted a comprehensive non-targeted metabolomics analysis of their serum samples. First, we used PLS-DA to assess the overall circulating metabolic profiles of the two patient groups. As shown in Figure 2A, the CRSwA group (red dots) and the CRSwNP-only group (purple dots) formed distinct clusters in the scores plot, revealing significant systemic metabolic phenotypic differences between the two clinical subtypes. To ensure the reliability of the model, we performed a 200-cycle permutation test. The results (Figure 2B) showed that the model's R2 value (0.8888) was substantially higher than its Q2 value (0.2309), and both metrics outperformed the random models, confirming the model's robustness and indicating that the observed inter-group differences were statistically significant.

To identify the key metabolites driving this systemic variance, we performed a volcano plot analysis. As depicted in Figure 2C, we identified a total of 66 serum metabolites with significantly different abundances between the two groups, of which 34 were significantly upregulated (red) and 32 were significantly downregulated (blue) in the CRSwA group. A circular heatmap (Figure 2D) provided a more intuitive visualization of the expression patterns of key differential metabolites, showing that lipids and their derivatives constituted the primary category of differing compounds, followed by amino acids and polypeptides. This is highly consistent with the metabolic demands of inflammation and immune regulation in CRSwA.

To explore the potential link between systemic circulating metabolites and the local immune microenvironment of nasal polyps, we analyzed the correlations between the differential serum metabolites and the infiltration abundances of 22 types of immune cells in tissue samples. The correlation heatmap (Figure 2E) revealed a complex regulatory network between the systemic metabolic state and local immune responses. Notably, and consistent with the tissue metabolomics results, several key lipid mediators (e.g., FAHFA 40:3 and PE 36:3e) exhibited significant positive correlations with the abundances of various immune cells, including M2 macrophages and eosinophils. This finding has significant biological implications, suggesting that the lower airways may release metabolites into the circulation, which in turn remotely regulate or reflect the composition and functional state of local immune cells in nasal polyps.

To understand the biological significance of these systemic metabolic changes, we performed KEGG pathway enrichment analysis on the differential metabolites. The results (Figure 2F) indicated that these metabolites were significantly enriched in several key pathways, including glycerophospholipid metabolism, choline metabolism, retrograde endocannabinoid signaling, and autophagy. The enrichment of glycerophospholipid and choline metabolism in serum, together with tissue lipid mediator signals, points to altered membrane lipid remodeling and ether phospholipid/plasmalogen turnover—lipid classes implicated in oxidative stress buffering and epithelial barrier dynamics in airway disease. FAHFA species (e.g., FAHFA 40:3) and ether lipids (e.g., PE 36:3e) that correlate with M2 macrophages/eosinophils may reflect systemic cues that reinforce Type 2 immune programming.

Finally, given the ease of access to serum samples, we evaluated the potential of these differential metabolites as non-invasive diagnostic biomarkers. We constructed a logistic regression model and plotted its Receiver Operating Characteristic (ROC) curve. As shown in Figure 2G, the model achieved an Area Under the Curve (AUC) of 0.8322, demonstrating good to excellent diagnostic performance. This indicates that detecting a specific combination of serum metabolites holds promise for effectively distinguishing between the CRSwNP with asthma and CRSwNP-only subtypes.

In summary, this study, through the analysis of serum samples, not only revealed the unique systemic metabolic dysregulation characteristic of patients with CRSwNP and comorbid asthma but also, for the first time, established a complex association between circulating metabolites and the local tissue immune microenvironment. These findings provide crucial insights for further understanding the pathogenesis of CRSwA and for developing novel diagnostic markers.

### Characterizing the shared metabolome linking local tissue pathology to systemic circulation

To investigate the relationship between local and systemic metabolism, we first identified a core set of metabolites consistently present in both biological compartments. We found 75 metabolites that were reliably detected across all paired tissue and serum samples, which we define as the “shared metabolome.” This group represents the fundamental metabolic bridge connecting the local inflammatory site with the systemic circulation. The subsequent cross-compartment analyses in this section are focused exclusively on this defined set of 75 shared metabolites.

**Figure 3:**
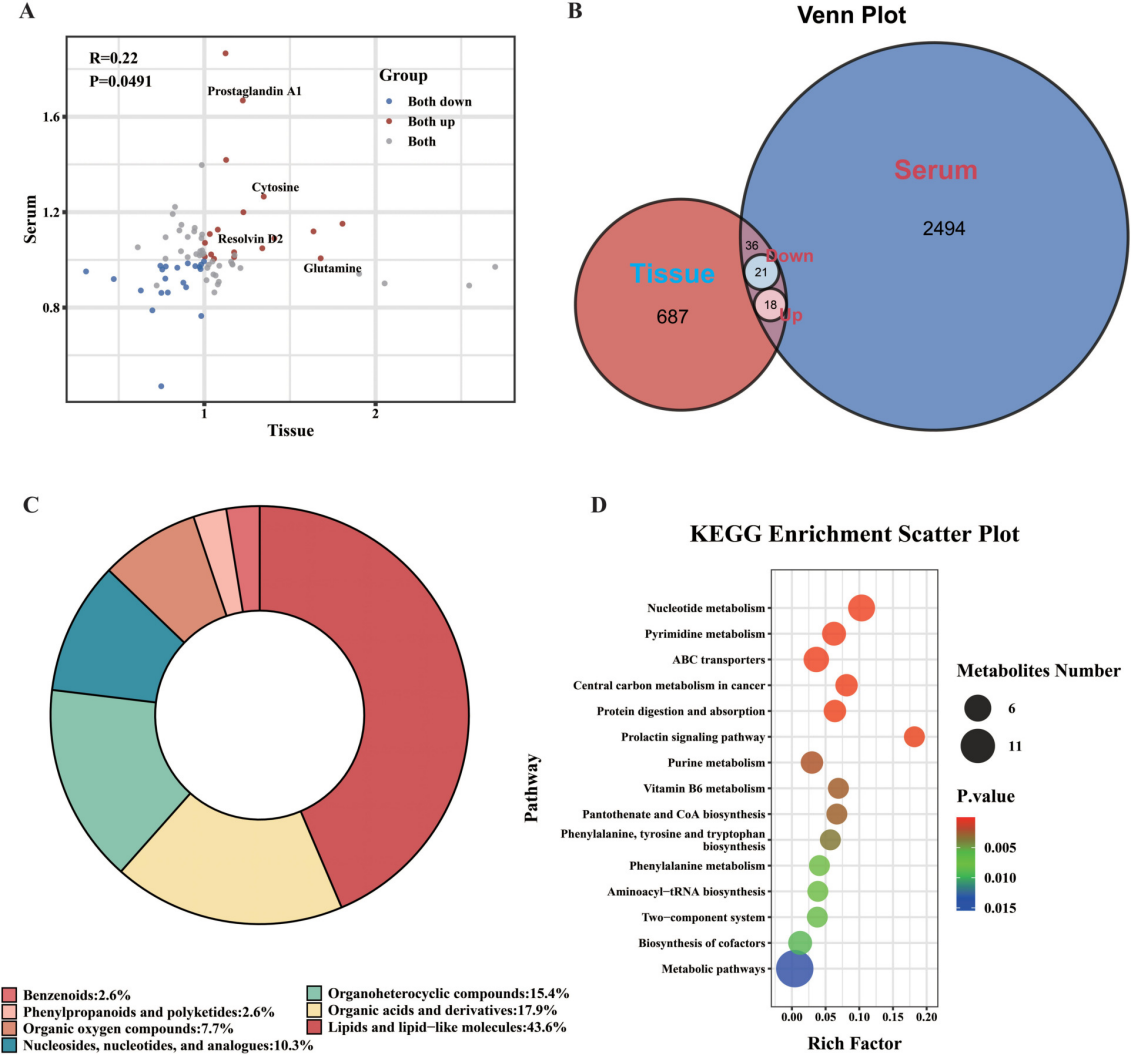
Characterization of the shared metabolome linking nasal polyp tissue and serum. All analyses in this figure are based on 75 metabolites consistently detected in both tissue and serum. **(A)** Correlation of log2(Fold Change) for the 75 shared metabolites. The plot compares fold changes (CRSwA vs. CRSwNP) between tissue (x-axis) and serum (y-axis). Red and blue points highlight metabolites with a concordant direction of change. Four metabolites that are statistically significant in tissue are labeled. The Pearson correlation (R) and *p*-value are shown. **(B)** Venn diagram of shared differential metabolites; 75 total, including 39 with significant, directionally concordant regulation across compartments (21 downregulated, 18 upregulated). **(C)** Chemical class distribution of the 75 shared metabolites. **(D)** KEGG pathway enrichment analysis of the 75 shared metabolites. Bubble size corresponds to the number of metabolites in the pathway, and color intensity represents the statistical significance (*p*-value).

We began by assessing the collective behavior of this shared metabolome by correlating the log2(fold changes) (CRSwA vs. CRSwNP) between tissue and serum. This analysis revealed a weak but statistically significant positive correlation (Pearson's R = 0.22, *P* = 0.0491; Figure 3A). This finding suggests that, as a whole, the metabolic network connecting the two compartments exhibits a non-random, coordinated response, although the magnitude of change differs substantially. Within this shared set, we highlighted metabolites with a concordant direction of change (Figure 3A, red and blue points) and noted that key molecules significantly altered in tissue, such as Glutamine and Resolvin D2, showed a consistent but non-significant trend in serum.

Chemical classification of the 75 shared metabolites showed a predominance of lipids and lipid-like molecules (43.6%), followed by organic acids and derivatives (17.9%) (Figure 3B). We then performed KEGG pathway enrichment on this entire 75-metabolite set to characterize the foundational metabolic network linking the compartments. The analysis highlighted pathways such as “Nucleotide metabolism,” “Purine metabolism,” and “Pyrimidine metabolism” (Figure 3C).

It is crucial to distinguish this finding from the pathway analyses of the most significantly altered metabolites within each compartment. The pathways enriched from the top differential metabolites in tissue (Figure 1E) or serum (Figure 2F) (e.g., glycerophospholipid metabolism) reflect the most intense pathological responses occurring in those specific environments. In contrast, the pathways enriched from the entire shared metabolome represent the nature of the underlying metabolic network that is constitutively active across both compartments. The difference in these enrichment patterns is therefore expected and biologically informative, distinguishing the pathways of acute pathological response from those of the fundamental local-systemic connection.

### A metabolic-immune axis governs local and systemic inflammation in with comorbid asthma

**Figure 4:**
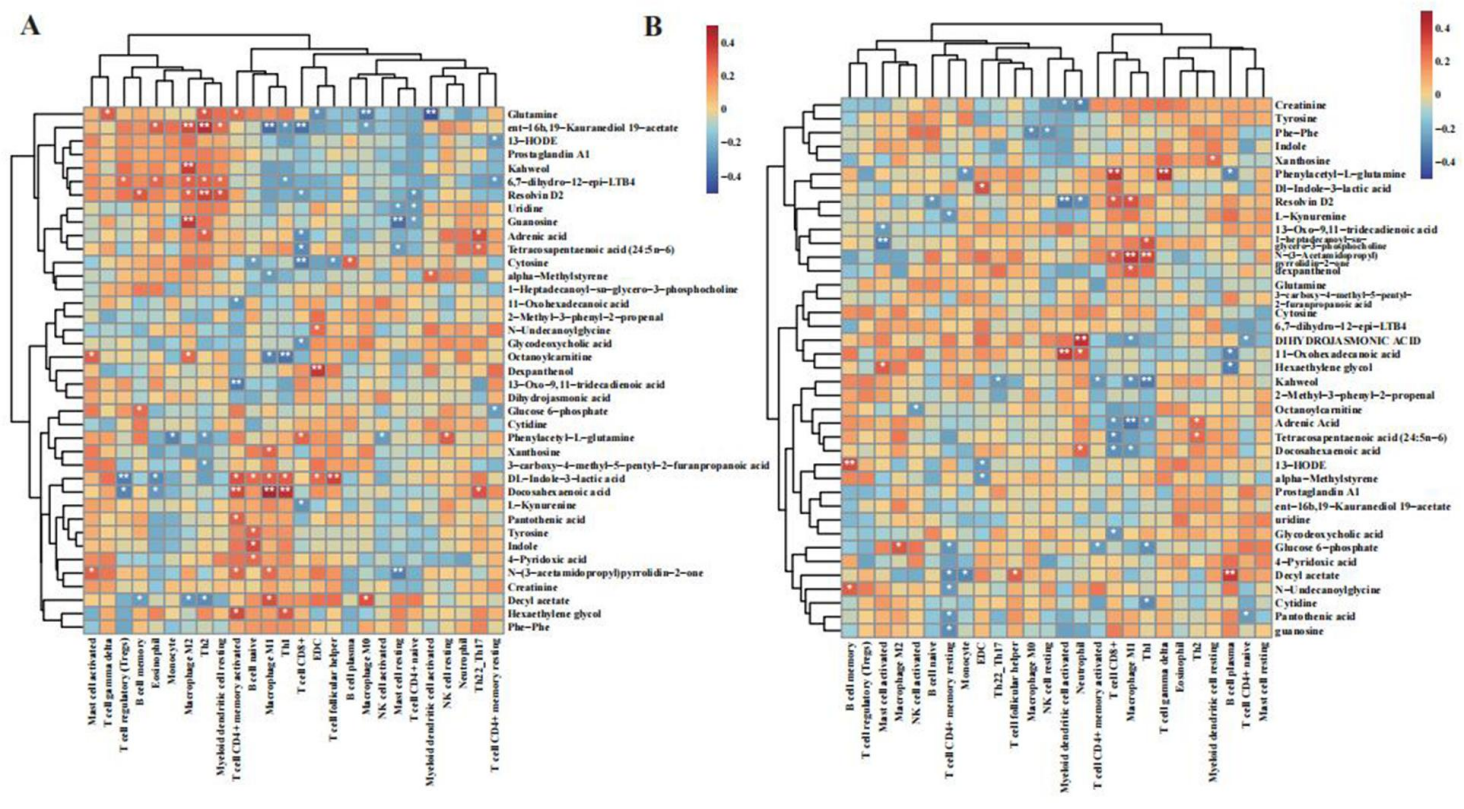
Integrated metabolic–immune landscape in CRSwA across tissue and serum. **(A)** Tissue-level associations between key metabolites and immune features (CIBERSORT and ssGSEA). Heatmap shows Spearman *ρ*; only |*ρ*| ≥ 0.3 and FDR q < 0.05 are annotated with asterisks. Selected Type I (Th1/M1/B-cell) and Type II (eosinophil/mast cell/M2) axes are indicated for orientation. **(B)** Serum–tissue cross-compartment correlations linking circulating metabolites with tissue immune features (Spearman; |*ρ*| ≥ 0.3 and FDR q < 0.05 considered significant). All *p*-values are two-sided; multiple testing controlled by Benjamini–Hochberg FDR at 5%. Full metabolite names, IDs, and identification levels (MSI) are provided in Supplementary Material.

To elucidate the interaction mechanisms of the immune microenvironments in the upper and lower airways in CRSwA, we conducted a multi-omics integrative analysis to deeply investigate the co-regulated metabolites in tissue and serum and their intrinsic association with the immune microenvironment. At the local tissue level (Figure 4A), metabolic reprogramming was a key driver of distinct immune response types. The Type I immune response was closely associated with the significant activation of tryptophan metabolism. Its key products, DL-indole-3-lactic acid and indole, showed a significant positive correlation with the abundance of M1 macrophages, Th1 cells, and B cells—core components of Type I immunity—thereby revealing the central role of tryptophan metabolism in driving cell-mediated Type I inflammation. Correspondingly, the Type II allergic inflammatory response was predominantly regulated by lipid metabolism. Specific lipid molecules, such as 6,7-dihydro-12-epi-LTB4 and Resolvin D2, were highly correlated with eosinophils and activated mast cells, indicating that lipid metabolic pathways like the arachidonic acid pathway are a core mechanism driving eosinophil infiltration and mast cell activation.

At the systemic level (Figure 4B), changes in serum metabolites precisely mirrored and potentially exacerbated the local immune dysregulation in the tissue. Serum metabolites, such as phenylacetyl-L-glutamine, exhibited a strong positive correlation with activated CD8+ memory T cells and the Th1 inflammatory response within the tissue. This suggests that systemic dysregulation of amino acid and phospholipid metabolism is tightly coupled with the exacerbation of local Type I/neutrophilic inflammation. Similarly, serum lipids like adrenic acid were also strongly correlated with eosinophil abundance, corroborating that systemic lipid dysregulation is a key factor in enhancing local Th2 inflammatory responses.

Taken together, the highly consistent lipid metabolic signatures in both tissue and serum revealed a bidirectional regulatory mechanism: lipid mediators produced in local tissue can enter the systemic circulation to influence distant airways, while circulating lipid precursors can, in turn, be transported to the lesion site to further amplify local Type II inflammation. This metabolite-mediated “local-systemic-local” interplay provides a novel perspective on the synergistic inflammation of the upper and lower airways in CRSwA, highlighting the central bridging role of metabolic networks in connecting local and systemic immune responses.

## Discussion

This study provides a comprehensive multi-omics analysis that, for the first time, systematically bridges the gap between local sinonasal inflammation and systemic metabolic dysregulation in patients with CRSwNP and CRSwA. Our key findings reveal that CRSwA is characterized by a distinct metabolic signature dominated by lipids and their derivatives, which is remarkably consistent between nasal polyp tissue and serum. This shared metabolic profile is intricately linked to specific immune cell infiltration patterns, defining a “metabolic-immune axis” that governs the pathological interplay between the upper and lower airways. This concept builds upon recent work highlighting metabolic reprogramming as a hallmark of chronic inflammatory diseases, including CRS (14, 15). These results not only deepen our understanding of the “unified airway” hypothesis from a metabolic perspective but also identify potential non-invasive biomarkers and therapeutic targets.

A central finding of our work is that metabolic reprogramming within the local tissue microenvironment is a primary driver of the Th2-polarized inflammation characteristic of CRSwA. We identified a significant upregulation of specialized pro-resolving mediators (SPMs) like Resolvin D2 and Lipoxin A4, which, paradoxically, correlated positively with Th2 effector cells such as eosinophils and M2 macrophages. While SPMs are classically known to resolve inflammation, their accumulation in chronic Th2 diseases suggests a state of “unresolved inflammation” or a dysregulated resolution process (16). The mechanistic role of specific SPMs like Resolvin E1 in actively suppressing key inflammatory pathways in allergic airway inflammation has been well-documented (17). This phenomenon of failed resolution is a recognized feature of severe asthma, where pro-resolving lipid mediator pathways are dysregulated, leading to a deficit in resolution signals relative to potent pro-inflammatory ones (17). In Th2-dominant contexts, these lipid mediators may be insufficient to counteract the inflammation, or their signaling pathways may be impaired, thus failing to terminate the persistent inflammatory response (18). Paradox and mechanistic model of elevated SPMs in a Th2-dominant, unresolved state: The increase of SPMs (e.g., Resolvin D2, Lipoxin A4) alongside Type 2 inflammation in CRSwA suggests production exceeds efficacy. Likely contributors are compensatory biosynthesis under chronic IL-4/IL-13 signaling (upregulating ALOX15/12, COX-2), coupled with limited resolution competence due to SPM receptor downregulation/desensitization (ALX/FPR2, ChemR23, GPR32), downstream signaling defects (PPAR*γ*–MerTK–efferocytosis), and accelerated SPM inactivation. Concurrent overproduction of pro-inflammatory eicosanoids (LTB4, PGD2) creates an unfavorable SPM-to-eicosanoid balance, and compartmentalization may restrict bioactive SPM pools. These factors, reported in severe asthma, likely explain the observed correlations and indicate a dysregulated resolution program. This model predicts that receptor functionality and mediator ratios—not absolute SPM levels—govern resolution in CRSwA. Additionally, the elevation of glutamine aligns with its known role in fueling immune cells. Recent studies confirm that amino acid metabolism, particularly the glutamine pathway, is a critical checkpoint for Th2 cell differentiation and the execution of their effector functions in allergic settings (19, 20). Our data thus paint a picture where local metabolic shifts in both lipid and energy pathways converge to create and sustain the distinct Th2 immune milieu of CRSwA.

Crucially, our study demonstrates that this localized metabolic dysregulation is not an isolated event but is mirrored systemically. The high concordance (Pearson's R = 0.99) between tissue and serum metabolic profiles is a striking discovery, providing robust evidence that circulating metabolites are a direct reflection of local pathological processes. This finding extends the unified airway concept, which has traditionally focused on cellular and cytokine trafficking, to the metabolic level (5, 6). The ability of our serum metabolite-based logistic regression model to distinguish CRSwA from non-asthmatic CRSwNP with high accuracy (AUC = 0.8322) underscores the clinical potential of this systemic signature. This aligns with recent metabolomic studies that have successfully identified circulating biomarkers capable of stratifying asthma endotypes and predicting therapeutic responses (21). Circulating lipids and amino acids, which were among the most discriminative features, may serve as accessible, non-invasive biomarkers for diagnosing CRSwA, stratifying patients, or monitoring disease activity, a significant advancement over more invasive diagnostic methods (8).

Our integrated analysis further allowed us to propose a “metabolic-immune axis” that operates across both local and systemic compartments, offering a novel mechanistic framework for the CRSwA phenotype. We identified two distinct arms of this axis: a tryptophan-metabolism-driven Type I immune response and a lipid-metabolism-driven Type II response. The association between tryptophan metabolites and Type I cells is consistent with the role of the aryl hydrocarbon receptor (AhR), a key sensor of these metabolites. Recent reviews have highlighted AhR's dualistic role in immunity, where its activation can either promote tolerance or exacerbate inflammation depending on the specific ligand and cellular context, a critical factor in allergic diseases (22). Conversely, the strong link between arachidonic acid pathway derivatives and Type II effector cells confirms the central role of lipid mediators. Eicosanoids, such as leukotrienes and prostaglandins, are now understood not just as inflammatory agents but as critical orchestrators that shape the entire lifecycle of Type 2 immune responses, from initiation to chronicity (23). Furthermore, immune cells possess distinct metabolic programs; for instance, the metabolic switch towards fatty acid oxidation is a defining characteristic of anti-inflammatory M2 macrophages, which are prevalent in Type 2 settings (24). The fact that these metabolic-immune links were observable in both tissue and serum suggests a bidirectional “local-systemic-local” interplay. We hypothesize that lipid mediators from the nasal polyps enter circulation, while systemic metabolic substrates, such as circulating fatty acids from diet or adipose tissue, are actively taken up by airway immune cells to fuel local inflammatory responses, creating a self-amplifying loop (25). This model provides a compelling explanation for the synergy between upper and lower airway inflammation in CRSwA.

Despite the novelty of these findings, our study has several limitations. First, its cross-sectional design allows us to establish strong associations but not causality. Longitudinal studies are needed to determine whether metabolic changes precede or result from immune dysregulation. Second, while our multi-omics analysis provides a comprehensive overview, the functional consequences of these metabolic alterations were not directly tested. Future *in vitro* and *in vivo* experiments using cell cultures or animal models are required to validate the specific roles of identified metabolites in modulating immune cell function. Finally, the study was conducted at a single center with a moderate sample size, which may limit the generalizability of our findings. Multi-center studies with larger and more diverse cohorts are warranted to confirm these results. Future work will quantify SPM receptor expression and desensitization markers in nasal polyp myeloid/epithelial subsets, assess PPAR*γ*–MerTK signaling and macrophage efferocytosis, and perform targeted lipidomics to derive SPM-to-eicosanoid ratios (RvD2/LXA4 vs. LTB4/PGD2). *Ex vivo* explant and epithelial–immune co-culture assays will test whether SPMs or pathway modulators restore resolution phenotypes. These experiments will directly evaluate the proposed model and its causal relevance.

## Conclusions

In conclusion, our study deciphers a highly conserved metabolic signature that connects local tissue inflammation with systemic circulation in patients with CRSwNP and comorbid asthma. We demonstrate that specific metabolic pathways, particularly those involving lipids and tryptophan, are tightly coupled with distinct immune endotypes, forming a metabolic-immune axis that drives the disease's pathophysiology. This work repositions metabolic dysregulation as a central hub in the unified airway disease model and opens promising new avenues for the development of serum-based diagnostics and **metabolically-targeted therapies for this challenging clinical condition.

## Data Availability

The datasets presented in this study can be found in online repositories. The names of the repository/repositories and accession number(s) can be found in the article/Supplementary Material.
