# Supplementary material for: A shared metabolic-immune axis links local and systemic inflammation in chronic rhinosinusitis with comorbid asthma
Source: Front Allergy. 2026 Jan 29;6:1700350. doi: 10.3389/falgy.2025.1700350 (PMC12894330; doi:10.3389/falgy.2025.1700350)
Supplement: Supplementary file 1 [file Table1.docx]

**Supplementary Table 1. Demographic characteristics of the cohorts**

| **Characteristics** | **CRSwA**  **（n=22）** | **CRSwNP**  **（n=40）** | ***P* value** |
| --- | --- | --- | --- |
| **Male, n (%)** | 13 (59.091) | 31 (77.500) | 0.127 |
| **Age (years)** | 48.864±12.334 | 48.200±13.885 | 0.852 |
| **smoker, n (%)** | 8 (36.364) | 12 (30.000) | 0.608 |
| **Revision surgery, n (%)** | 7 (31.818) | 8 (20.000) | 0.298 |
| **AR, n (%)** | 14 (63.636) | 16 (40.000) | 0.075 |
| **Serum total IgE (kU/l)** | 120.500 (35.050, 255.750) | 47.900 (20.775, 198.250) | 0.139 |
| **VAS, M (Q1, Q3)** | 7.000 (6.000, 7.750) | 6.000 (4.000, 7.250) | 0.101 |
| **SNOT-22, M (Q1, Q3)** | 35.500 (27.250, 39.500) | 27.500 (17.750, 37.250) | 0.096 |
| **LM CT score, M (Q1, Q3)** | 8.500 (7.000, 10.000) | 8.000 (6.000, 9.000) | 0.273 |
